# Microbiological Safety and Functional Properties of a Fermented Nut-Based Product

**DOI:** 10.3390/foods13193095

**Published:** 2024-09-27

**Authors:** Giulia Tabanelli, Chiara Montanari, Ana M. Gómez-Caravaca, Elixabet Díaz-de-Cerio, Vito Verardo, Fatemeh Shanbeh Zadeh, Lucia Vannini, Fausto Gardini, Federica Barbieri

**Affiliations:** 1 Department of Agricultural and Food Sciences, University of Bologna, 40127 Bologna, Italy; giulia.tabanelli2@unibo.it (G.T.); fatemeh.shanbehzade2@unibo.it (F.S.Z.); lucia.vannini2@unibo.it (L.V.); fausto.gardini@unibo.it (F.G.); federica.barbieri16@unibo.it (F.B.); 2 Interdepartmental Centre for Industrial Agri-Food Research, University of Bologna, 47521 Cesena, Italy; 3 Department of Analytical Chemistry, Faculty of Sciences, University of Granada, Avd. Fuentenueva s/n, 18071 Granada, Spain; anagomez@ugr.es; 4 Biomedical Research Center, Institute of Nutrition and Food Technology ‘José Mataix’, University of Granada, 18071 Granada, Spain; vitoverardo@ugr.es; 5 Department of Nutrition and Food Science, University of Granada, Campus of Cartuja s/n, 18071 Granada, Spain; elixabet.dzdecerio@gmail.com

**Keywords:** vegan product, cheese analogues, lactic acid bacteria, bioprotective cultures, *Listeria monocytogenes*, *Escherichia coli*, *Salmonella* Enteritidis, phenolic content

## Abstract

Fermented nut-based products, obtained after soaking and fermentation, are gaining increasing interest as animal food substitutes because of ethical, environmental and health reasons. In these products, Lactic Acid Bacteria (LAB) perform the fermentation, leading to matrix acidification and contributing to controlling spoilage and pathogenic microbiota. In this work, LAB strains isolated from an artisanal product and combined with a commercial strain were added as starter cultures during nut soaking to produce a cheese-like fermented plant-based product. Three different LAB consortia were used in challenge tests at laboratory scale against *Listeria monocytogenes*, *Escherichia coli* or *Salmonella* Enteritidis, inoculated in nuts at 5 log CFU/g, and monitored for pathogen survival and matrix acidification. The combination of *Lactiplantibacillus plantarum* 82 and *Leuc. carnosum* 4010 resulted in faster acidification (pH value < 4.4 after 18 h instead of 48 h) and the reduction of target pathogens; *L. monocytogenes* was already absent after seven days from production, and the counts of *E. coli* or *S.* Enteritidis were lower with respect to other samples. Thus, this microbial consortium was used for a pilot-scale production in which, beyond safety, the fermented plant-based product was also characterized for aroma profile and phenolic compounds, parameters that are known to be affected by LAB fermentation. The results showed an enhancement of the aroma profile, with an accumulation of molecules able to confer cheese-like notes (i.e., acetoin and diacetyl) and higher phenolic content, as well as the presence of compounds (i.e., phenyllactic acid and hydroxyphenyllactic acid) that could exert antimicrobial activity. This study allowed us to set up a guided fermentation for a cheese-like vegan product, guaranteeing safety and improving aromatic and functional features.

## 1. Introduction

In recent years, the increasing attention towards “green”, nutritional, and ethical aspects of foods has induced a higher consumer interest in vegan, vegetarian, and flexitarian diets, with about 5% of people in Western countries following a plant-based nutrition [1,2]. Therefore, the renewed interest in plant-based dairy and meat alternatives has opened great opportunities for food industries to fulfil consumer demand, entering new markets which are estimated to reach a value of USD 422.26 million by 2026 [3].

Products obtained from soy (i.e., tofu), rice, or other cereals are the most commonly available cheese and meat substitutes. Recently, there has been a growing popularity of artisanal fermented products derived from various types of nuts, such as cashews, macadamias, almonds, and others [4]. These plant-based dairy alternatives can be healthier compared to products of animal origin in terms of lower saturated fat and sugar, with similar protein content [1]. The mixture of these nuts, after soaking and water addition, undergoes a spontaneous fermentation, which is often performed at the domestic level. The fermentation process in which specialized microorganisms grow in these products increases their nutritional and functional characteristics (reduction of antinutritional compounds, increase of nutrient bioavailability, potential use of probiotic species) as well as the organoleptic features (accumulation of aroma compounds), being able also to enhance their microbiological safety [3]. Indeed, under favourable conditions, vegetables can undergo spontaneous lactic acid fermentation, resulting in acidification within the food matrix and alterations in abiotic conditions and contributing to controlling the Gram-negative bacteria, which are particularly vulnerable to fermentation effects [5,6]. However, considering the domestic or artisanal nature of the fermented nut-based products and the frequent absence of specific starter cultures to drive the process, the safety and quality of the artisanal plant-based products are not always assured [3]. Additionally, traditional technological knowledge for fermenting nuts to produce cheese substitutes is lacking, and scientific documentation in this field is still scarce, mainly because these products are relatively new and innovative [7].

From a food safety perspective, all the process steps have inherent risks, together with the ingredients and raw materials used in the manufacturing. In fact, low-moisture foods, such as nuts, are generally considered less susceptible to the growth of foodborne pathogens, but the long-term survival of the same bacteria in these products is well documented [8,9]. For these reasons, the control of microbiological quality of the raw materials is of major importance in fermented nut-based foods, with pH, acidity, and high salt content being the main parameters that can inhibit undesirable microorganisms.

From this perspective, a strictly guided fermentation using specific functional Lactic Acid Bacteria (LAB) able to counteract spoilage or pathogenic microbiota is a key factor to avoid, or at least reduce, the biological risks associated with these innovative products [10]. In fact, the fermentation process, one of the most ancient and diffused strategies of food stabilization, can be applied in bio-protection, obtaining high added-value foods and guaranteeing the innovative products’ safety [7,11]. LAB fermentation agents can be endowed with bio-protection features thanks to their aptitude to compete with spontaneous microbiota by the production of specific metabolites (i.e., bacteriocins, organic acids, etc.), assuring food safety and extending product shelf life while maintaining the foods’ nutritional and sensorial properties [12,13,14]. Moreover, fermentation affects the nutritional value of the matrices with the accumulation of secondary metabolites with bioactive features (exopolysaccharides, short-chain fatty acids, bioactive peptides) and the degradation of antinutritional factors, leading to an improvement in the bioavailability of essential nutrients [11,15]. Specifically, fermentation can improve the content of phenolic compounds and change their profile due to the release of bound phenolic compounds as a consequence of the degradation of the cell wall’s structure by microbial enzymes [16]. Thus, the fermented product is characterized by improved overall antioxidant capacity [17], as well as the bioavailability of polyphenols, which have been demonstrated to provide several benefits for human health, such as reduction in the incidence of some degenerative diseases, the reduction in risk factors of cardiovascular diseases, and the enhancement of antioxidant, anti-allergenic, anti-inflammatory, and antimicrobial effects.

In addition, LAB fermentation can positively affect the flavour of foods, leading to a higher content of desirable volatile compounds, which results in higher aroma complexity and better sensorial characteristics [18,19,20].

Tabanelli et al. [21] studied the spontaneous fermentation that occurs during the manufacture of a home-made cashew-nut fermented cheese analogue intended for vegan consumers. A crucial aspect for the commercialization of these products is the definition of process risk points and the implementation of microbial challenge tests to evaluate the safety aspects associated with its production, especially in relation to the presence of *Enterobacteriaceae* (such as *Escherichia coli* and *Salmonella* spp.) and *Listeria monocytogenes*, which represent the major microbiological hazards linked to this product. The presence of these microorganisms can be due to contaminated raw materials and inadequate sanitizing of processing equipment. The main obstacle to their growth and survival is represented, together with a strictly controlled refrigerated storage (0–4 °C), by a fast pH drop to under 4.4 during fermentation and the maintenance of a low pH value during shelf life.

The aim of this work was to assess the microbial safety of a vegan cheese analogue in relation to the LAB strains used for fermentation. In particular, cashews and almonds were soaked, ground and then fermented in order to obtain a spreadable cheese analogue (Quark style). Two different productions were considered. The first was carried out at laboratory level, and three different combinations of LAB starter cultures were used. All these combinations were challenged by inoculating *Listeria monocytogenes* Scott A, *Escherichia coli* 555 and *Salmonella* Enteritidis 155 and evaluating the survival/growth of the pathogens. The second was carried out in an industrial pilot plant, using the microbial consortium with the better performances in the previous step and challenged against the same pathogens. In addition, the fermented cheese analogue obtained in the pilot plant production not inoculated with pathogens was also characterized, taking into consideration sensorial and functional aspects. In fact, the compounds responsible for the aromatic characteristics of the product were detected by using a GC-MS-SPME analytical protocol. Then, the composition of phenolic fraction was investigated by UPLC with the aim to highlight the possible effect of fermentation on the content of molecules with important functional value.

## 2. Materials and Methods

### 2.1. Microbial Strains

The LAB strains used as starters for nut fermentation were *Leuconostoc mesenteroides* (LmV1) and *Pediococcus pentosaceus* (PpV1) previously isolated from home-made fermented cashew products [21]; *Lactiplantibacillus plantarum* 82, belonging to the Microbial Culture collection of the Department of Agricultural and Food Science (University of Bologna); and the bioprotective commercial strain *Leuconostoc carnosum* 4010, isolated from vacuum-packed sliced ham [22] (Chr. Hansen A/S, Parma, Italy). These strains were stored in 20% (*w*/*v*) glycerol at −80 °C and pre-cultivated for 24 h at 30 °C in de Man, Rogosa, and Sharpe (MRS) broth (Oxoid, Basingstoke, UK).

The target strains used during challenge tests were *Listeria monocytogenes* Scott A, *Escherichia coli* 555 and *Salmonella* Enteritidis 155 (belonging to the Microbial Culture collection of the Department of Agricultural and Food Sciences, University of Bologna). The strains were maintained in a BHI medium (Oxoid, Basingstoke, UK) with 30% (*w*/*v*) glycerol at −80 °C and, before the experiments, pre-cultivated twice (37 °C for 24 h) in a BHI medium. For the trials, pathogen overnight cultures were centrifuged, washed, and resuspended in the same volume of sterile saline solution (0.9% *w/v* NaCl) to be inoculated at a level of about 5.5 log CFU/g in the raw material.

### 2.2. In Vitro Antagonistic Activity of LAB Starter Strains against L. monocytogenes ScottA, Escherichia coli 555 and Salmonella Enteritidis 155

The antibacterial activity of LAB strains was determined using the agar spot test and the well-diffusion assay described by Schillinger and Lücke [23]. The production of antimicrobial substances was confirmed by well-diffusion agar assay using filter-sterilized and neutralized cell-free supernatants and filter-sterilized, neutralized, and heat-treated (98 °C for 20 min) cell-free supernatants. To evaluate the sensitivity of the inhibitory substances to proteases, the well-diffusion agar assay was repeated after treatment of the cell-free supernatants with proteinase K (2 mg/mL, Sigma-Aldrich, Milano, Italy) and pepsin (1 mg/mL, Sigma-Aldrich) at 37 °C for 4 h. The results were expressed as diameter of the inhibition zone (mm) after the incubation period, and the data are the mean of three different experiments.

### 2.3. Challenge Test in Nut-Based Fermented Product at Laboratory Scale

**Figure 1:**
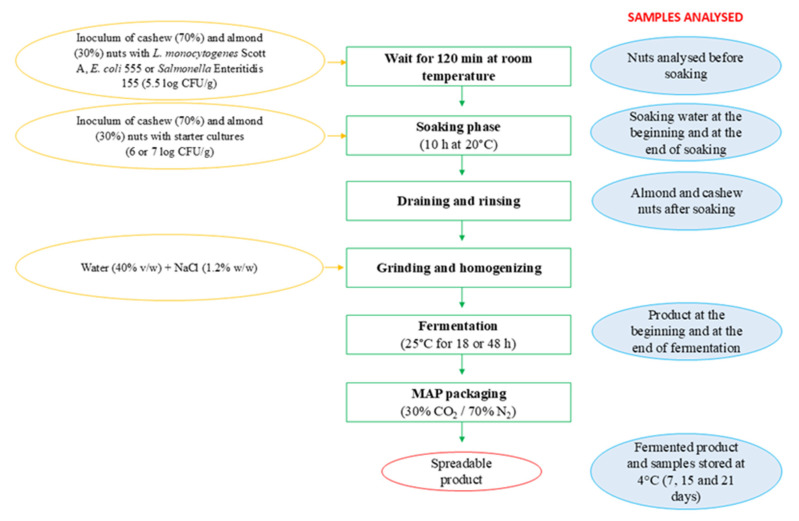
Flow sheet of fermented cashew and almond nut production during challenge test. The sampling points are also reported.

The cheese analogue was obtained by fermenting nuts. In particular, 6 kg of cashews (70%) and almonds (30%) were used (all provided by Euro Company Srl, Ravenna, Italy). The flow sheet of the production process is reported in Figure 1. Briefly, the nuts were soaked at 20 °C for 10 h in water added with LAB starter cultures. After, the nuts were drained and added with 40% (*w*/*w*) of fresh water and salt (1.2% *w*/*w*). Then, this mixture was ground with a mixer to obtain a homogeneous cream. The cream obtained was left to ferment at 25 °C until achieving a pH value lower than 4.4 (ranging from 18 to 48 h). The fermented spreadable product was portioned (80 g) and packaged under MAP (30% CO_2_ and 70% N_2_) in a high-oxygen-barrier plastic film and stored at 4 °C for 21 days.

Following the flow sheet in Figure 1, three different products were obtained, differentiated by the starter cultures used. In trial 1, the strains *Leuc. mesenteroides* (LmV1) and *P. pentosaceus* (PpV1) were inoculated at a concentration of 7 log CFU/mL and 6 log CFU/mL, respectively. In trial 2, an inoculum of 7 log CFU/mL of *Leuc. mesenteroides* LmV1 and 6 log CFU/mL of *Lpb. plantarum* 82 were used. In trial 3, the bioprotective commercial strain *Leuc. carnosum* 4010 was tested together with *Lpb. plantarum* 82 at a concentration of 7 log CFU/mL and 6 log CFU/mL, respectively. For each trial, four aliquots (approx. 1.5 kg) were obtained. The first was considered as a control with no pathogen inoculum. The remaining three aliquots were separately inoculated with *L. monocytogenes* Scott A, *E. coli* 555 or *S.* Enteritidis 155. The inoculum of pathogens was carried out directly in nuts approx. 120 min before the soaking phase to reach an initial concentration of approx. 5.5 log CFU/g. During production and storage, samples were collected at different sampling points, as reported in red in Figure 1. The challenge test trials were performed in duplicate, and samples were withdrawn in triplicate.

### 2.4. Pilot-Scale Production

A challenge test of fermented plant-based product was also carried out in a pilot plant, using the most promising starter cultures tested in the previous trials. Three batches of 10 kg each were inoculated with *L. monocytogenes* Scott A, *E. coli* 555 or *S*. Enteritidis at a concentration of approx. 5.5 log CFU/g. A fourth 10 kg batch not inoculated with pathogens was produced as a control. *Lpb. plantarum* 82 and *Leuc. carnosum* 4010 were added as starter cultures to the soaking water at a concentration of 6 log CFU/g and 7 log CFU/g, respectively. The process followed the same flow sheet reported in Figure 1 but included a treatment in hot water (85 °C for 10 min) of the nuts inoculated with pathogens before the soaking phase. The spreadable product was packaged under MAP (30% CO_2_ and 70% N_2_) in a high-oxygen-barrier plastic film and stored at 4 °C for 30 days to detect pathogen survival and quality parameters of the control batch.

### 2.5. pH, a_w_ and Microbiological Analyses

The a_w_ and pH were detected by using an Aqualab CX3-TE (Labo-Scientifica, Parma, Italy) and a pH-meter Basic 20 (Crison Instruments, Barcelona, Spain), respectively. These determinations were performed in triplicate.

Microbiological analyses were carried out in triplicate for each challenge test. Specifically, soaking water (1 mL) was aseptically transferred to 9 mL of 0.9% (*w*/*v*) NaCl sterile solution. For nuts and nut products, 10 g of samples were aseptically added in 90 mL of 0.9% (*w*/*v*) NaCl sterile solution and homogenized in a Lab Blender Stomacher (Seward Medical, London, UK) for 2 min. The resulting suspensions were serially diluted and plated onto selective media. Counts of lactobacilli were obtained by plating appropriate dilutions on MRS agar (Oxoid, Basingstoke, UK) incubated at 30 °C for 48 h in anaerobic conditions. *Enterobacteriaceae* were enumerated by pour plating in Violet Red Bile Glucose Agar (VRBGA, Oxoid) at 37 °C for 24 h. Enterococci were counted by surface-plating on Slanetz and Bartley medium (Oxoid) incubated at 44 °C for 24 h, while for the yeast counts, Sabouraud Dextrose Agar (Oxoid) added with 200 mg/L of chloramphenicol was used and incubated at 28 °C for 72 h.

The detection of the inoculated pathogens was performed by plate counting in the following selective media (all provided by Oxoid, Basingstoke, UK): Listeria Selective Agar Base (LSO) added with Selective Listeria Supplement for *L. monocytogenes*, incubating the plates at 30 °C for 48 h; Violet Red Bile Agar added with 4-Methylumbelliferyl-*β*-D-glucuronide for *E. coli*, incubating the plates at 37 °C for 24 h; and XLD medium for *S.* Enteritidis, incubating the plates at 37 °C for 24 h.

When *L. monocytogenes* and *S.* Enteritidis were under the detection limit, enrichment of the inoculated samples was performed. Regarding *L. monocytogenes*, 25 g of the product were homogenized for 120 s with 225 mL of Listeria Primary Selective Enrichment Medium (UVM I, Oxoid) and incubated in the Stomacher bag at 30 °C for 24 h. From this bag, 0.1 mL was transferred into 10 mL of Listeria Secondary Enrichment Medium (UVM II, Oxoid), while 1 mL was transferred into a 4.5 mL KOH sterile solution (KOH 2.5 g/L, NaCl 20 g/L), homogenized by vortex, and within one minute, sub-cultured on Listeria Selective Agar plates (Oxoid). After 24 h of incubation at 30 °C, 0.1 mL of inoculated Listeria Secondary Selective Enrichment Medium (UVM II) was spread onto Listeria Selective Agar plates, while 1 mL was transferred to a 4.5 mL KOH sterile solution, homogenized by vortex, and within one min, sub-cultured on Listeria Selective Agar plates (Oxoid). All plates were incubated at 30 °C for 48 h.

To assess the presence of *Salmonella*, 25 g of the sample were homogenized for 120 s with 225 mL of Buffered Peptone Water (Oxoid) and incubated in the Stomacher bag at 30 °C for 24 h. Then, 0.1 mL was transferred into 10 mL of Rappaport-Vassiliadis Enrichment Broth (RVS, Oxoid) and incubated at 42 °C. After 24 h, an aliquot of this suspension was streaked onto XLD and Bismuth Sulphite Agar (BSA, Oxoid) plates that were incubated at 37 °C for 24 h.

### 2.6. Volatile Profiles

Volatile compounds (VOCs) of the control batch obtained during pilot-scale process optimization were monitored by using a GC-MS coupled with a solid phase micro-extraction (GC-MS-SPME) technique, according to the method reported by Tabanelli et al. [21]. The compounds are reported as the ratio between each peak area and the area of an internal standard (4-methyl-2-pentanol). The unidentified compounds were not included, accounting for less than 3% of total peak area.

### 2.7. Extraction and Determination of Phenolic Compounds

Phenolic compounds of the control batch obtained during pilot-scale process optimization were studied. Samples from the pre-fermented product (nut cream obtained after soaking and grinding), the fermented product, and the fermented product after 15 days stored at 4 °C were extracted following the previous protocol described by Gómez-Caravaca et al. [24]. Afterwards, phenolic compounds contained in the extracts were analysed by an ACQUITY Ultra Performance LC system (Waters Corporation, Milford, MA, USA) coupled to an electrospray ionization (ESI) source operating in negative mode and a time-of-flight (TOF) mass detector (Waters Corporation, Milford, MA, USA). The column was an ACQUITY UPLC BEH Shield RP18 column (1.7 µm, 2.1 mm × 100 mm; Waters Corporation, Milford, MA, USA), and the separation was done at 40 °C by using the gradient conditions described by Verni et al. [25]. Data were processed by using MassLynx 4.1 software (Waters Corporation, Milford, MA, USA).

## 3. Results and Discussion

### 3.1. Antagonistic Activity of LAB Strains against L. monocytogenes Scott A, E. coli 555 and S. Enteritidis 155

The antibacterial activity of LAB starter strains against target pathogens was determined by measuring the inhibition halos in an agar spot test (Table 1). All the starter strains showed antibacterial activity when spotted on cultural media, with inhibition zones ranging from 11 to 15 mm, with only slight differences between the strains.

When neutralized cell-free supernatants were tested, only *Leuc. carnosum* 4010 maintained the inhibition activity, but only against *L. monocytogenes* Scott A. This strong anti-listerial capacity was also evident when neutralized cell-free supernatants were heat treated at 98 °C for 20 min or treated with pepsin, confirming the proteinaceous nature of the anti-listerial substance produced by this strain. On the other hand, this bacteriocin was sensitive to proteinase K, a proteolytic enzyme, in accordance with results previously reported [26]. The anti-listerial activity of *Leuc. carnosum* 4010 is well documented, and the production of two different class IIa bacteriocins (i.e., leucocin A and leucocin C) has been demonstrated [27,28,29]. The same authors reported that this strain can produce, in addition to anti-listerial leucocins, a class IId bacteriocin leucocin B, which can be active against *Leuconostoc* and *Weissella* [30]. Recently, the whole genome of this strain has been analysed, demonstrating leucocin-related gene clusters on the plasmid pLC4010-2 [31].

### 3.2. Challenge Test in Nut-Based Fermented Product at Laboratory Scale

The survival of *L. monocytogenes* Scott A, *E. coli* 555 and *S.* Enteritidis inoculated in the nuts before laboratory-scale productions was assessed during the process and storage of the fermented nut product and obtained using three LAB consortia. In addition, the evolution of the main microbial populations (LAB, enterobacteria), pH and a_w_ in the control not inoculated with pathogens was monitored. The results showed that the fermentation process and the refrigerated storage could contribute to reduce the survival and proliferation of the target pathogens, depending on the LAB starter used (Table 2).

In all the trials, the chosen starter strains were able to proliferate during the soaking phase (considered as a pre-fermentation), reaching concentrations of about 9 log CFU/g after 10 h. This LAB growth lowered the water pH at values between 4.91 (trial 1) and 4.61 (trial 3) due to the accumulation of organic acids. During soaking, no proliferation of the target pathogens was shown. After nut grinding and homogenization, LAB were at levels of about 7.8–8.0 log CFU/g. During the fermentation process, LAB grew more than 1 log unit and maintained high concentrations during the whole shelf life at refrigerated temperature.

This massive growth permitted levels to reach pH values of 4.4 in the fermented spreadable product. This pH value is considered the safe threshold for the growth of *L. monocytogenes*. Since nut-based fermented products usually have a_w_ values higher than 0.94, it is of primary importance to guarantee that the pH is always maintained below 4.4 during shelf life, as indicated also by the EU regulation 2073/2005 [32]. This pH value can be considered critical also for *Salmonella* and *E. coli*, even if the susceptibility of these species to pH can vary according to other environmental conditions [33,34].

This proper acidification was reached at different times depending on the starter used. In particular, a pH lower than 4.4 was assessed after 48 h of fermentation in trials 1 and 2, while only 18 h were necessary for the proper acidification of trial 3 (Table 2).

Concerning pathogens, their cell-count decreases during process and storage were more evident in trials 2 and 3. As far as trial 2, *L. monocytogenes* Scott A was absent in 25 g of sample after 15 and 21 days of storage, while *S.* Enteritidis was not qualitatively detected only at the end of storage (21 days). At the same sampling time, *E. coli* was below the detection limit. The reduction of the target pathogens was more evident in trial 3, where the commercial starter *Leuc. carnosum* 4010 was able to kill listeria cells from the earliest production phases, suggesting an effective antimicrobial activity of this strain towards this Gram-positive pathogen also in this kind of product. It is well known that this strain produces leucocin A and leucocin C with anti-listerial action [27,28,29]. On the other hand, the Gram-negative pathogens (*E. coli* 555 and *S.* Enteritidis 155) were more persistent, especially during fermentation and early storage. Different studies regarding the survival of these pathogens during processing and storage of fermented vegetables demonstrated that, even if environments are not favourable to support their growth, pathogens can persist in the brines for most of the fermentation and during storage, according to salt concentration, pH, redox potential and additives [35,36,37]. This long-term survival of the pathogen species is also well documented in low-moisture foods, which are generally considered less susceptible to the growth of foodborne pathogens. In recent years, outbreaks linked to different nuts or nut products have increased, caused mainly by *Salmonella* spp., *Bacillus cereus*, *Cronobacter sakazakii* (formerly *Enterobacter sakazakii*), *Clostridium* spp., *Escherichia coli* O157:H7, and *Staphylococcus aureus* [9]. An outbreak caused by fermented cashew nut products contaminated with enterobacteria has been reported in the United States, with 30 people involved (of whom about 20% were hospitalized) [38,39]. Concerning other microbial groups, enterococci and yeasts were always under the detection limits in all the trials.

### 3.3. Pilot-Scale Production: Process Optimization and Challenge Test

**Figure 2:**
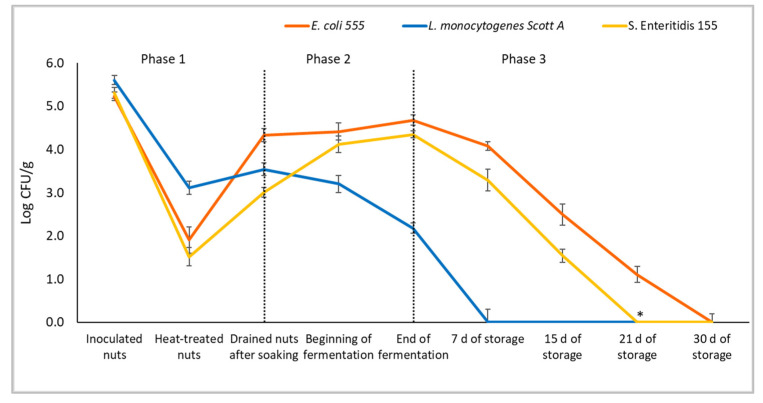
Microbial counts of inoculated pathogens during pilot-scale production optimization trial. The graph is divided into three phases: phase 1 includes nuts after treatment in a hot water bath and after the soaking phase, phase 2 comprises spreadable samples during fermentation and phase 3 includes spreadable products during storage. The presence of an asterisk indicates that *S.* Enteritidis was present in 25 g of sample.

The microbial consortium formed by *Leuc. carnosum* 4010 and *Lpb. plantarum* 82 was chosen for pilot-scale process optimization due to its acidification performances and ability to counteract the growth and survival of deliberately inoculated pathogens, enhancing the product’s hygienic quality and limiting the possible risks associated with its consumption. To design a proper process for this spreadable cheese analogue, nuts (deliberately inoculated with pathogens or not) were treated in a hot water bath (85 °C for 10 min) before soaking to study the possible use of this process phase to reduce the cell load of undesirable microbiota associated with the raw materials and the inoculated pathogens. The starter cultures confirmed the same behaviour and performances highlighted in trial 3 of the laboratory-scale challenge test, resulting in the same pH drop within 18 h of fermentation. The data regarding pathogen growth or survival during production and storage are shown in Figure 2. As observed, the applied thermal treatment was not able to completely inactivate the pathogens, probably because of a non-homogeneous distribution of heat in the nut mass and the internalization of microbial cells into nut pores. However, a cell load reduction of about 3 log units for *E. coli* 555 and *S.* Enteritidis and 2 log units for *L. monocytogens* Scott A was achieved. The latter was not able to grow during soaking and decreased during fermentation, probably due to the anti-listerial activity of *Leuc. carnosum* 4010, as already assessed during the previous challenge tests. Moreover, the population surviving (2 log CFU/g) in the fermented spreadable cheese analogue further decreased during the first days of storage, and this species was absent in 25 g of sample after seven days at 4 °C and in the following sampling times. On the other hand, *E. coli* 555 was able to grow during soaking, reaching a concentration of about 4.3 log CFU/g, then remaining quite constant during fermentation. Its cell load progressively decreased only during storage, confirming the high persistence rate of this species in acidified vegan fermented products. Similarly, *S.* Enteritidis 155 grew, even if slowly, during soaking and reached cell counts comparable to *E. coli* at the end of fermentation. During refrigerated storage, salmonella counts decreased more rapidly than *E. coli*. After 21 days, *S.* Enteritidis was qualitatively detected, even if the cell counts were below the detection limit, while after 30 days, it was absent in 25 g of the sample.

Probably, this is also due to the low NaCl content of the product (1.2% *w*/*w*), chosen to meet the nutritional need to reduce salt content in the human diet. However, it is noteworthy that even slightly higher amounts of NaCl could not be an effective hurdle to control enterobacteria. From this perspective, it is essential to optimize the possible treatment of raw materials to reduce the presence of possible Gram-negative microorganisms such as *E. coli* or *Salmonella*.

### 3.4. Analysis of Volatile Organic Compounds (VOCs) during Fermentation and Storage

The VOCs profiles of control samples (obtained in pilot-scale production with not-inoculated nuts) during fermentation and storage are reported in Table 3. The main aroma compounds that characterized this spreadable cheese analogue belonged to ketones, aldehydes, alcohols and acids, and their abundance increased during fermentation and storage. Among these chemical groups, alcohols were the most abundant compounds in the fermented samples, ethanol being the principal one. This molecule, which can derive from several pathways, such as pyruvate metabolism [40], increased during fermentation and storage. Other important alcohols were 1-hexanol, which derives from fat metabolism, 3-methyl-2-butanol, benzyl alcohol, and phenylethyl alcohol, which comes from the metabolism of amino acids and can bring sweet, fruity, or floral notes [41]. These molecules were present in the cream before fermentation as raw material components and as compounds produced by LAB activity during soaking, as shown by counts and the pH drop.

Acetic acid followed the same trend as ethanol and was the most important organic acid detected. This acid can be produced by heterofermentative bacteria, i.e., *Leuc. carnosum* starter, and/or can derive from LAB secondary metabolisms, which are more active when nutritional conditions become more astringent, as in the case of a lack of fermentable sugars.

Aldehydes were abundant before and after fermentation and underwent a decrease during storage, mainly due to a diminution of benzaldehyde. This compound is one of the fundamental components of almonds, and it is reduced to benzyl alcohol by microbial action, which increased consequently during storage. Among ketones, acetoin and diacetyl, deriving from pyruvate metabolism [42], were the most important. The presence of these compounds confers butter and dairy notes and can be important for the sensory characteristics of the product.

The progressive increase of ketones, alcohols, and acids during storage showed that secondary metabolic activities were present after the primary fermentation process and that aromatic equilibrium was needed for a product to rest at a low temperature.

### 3.5. Identification and Quantification of Phenolic Compounds by UPLC-MS during Fermentation and Storage

The phenolic compounds of the products obtained in pilot-scale production with nuts not inoculated with pathogens were analysed before and after the fermentation process and after 15 days of storage at 4 °C. A total of 31 phenolic and other polar compounds were identified in the samples (Table 4) according to their mass data and by comparing them with literature, the co-elution with commercial standards (when possible) and with several databases. Mass data, experimental and calculated *m*/*z* and error, mainly in source fragments and molecular formulae, were considered. Their quantification during fermentation and storage has been summarized in Table 5. Most of these compounds belong to the family of phenolic acids, flavonols and flavan-3-ols.

The total content of phenolic compounds increased by around 16% after the fermentation process, and it did not significantly vary after 15 days in storage. A few compounds were responsible for this increase: phenyllactic acid, hydroxyphenyllactic acid, dihydro-p-coumaric acid, dihydrophaseic acid-ribofuranosyl-glucopyranoside and p-coumaric acid hexoside pentoside isomer II.

Phenyllactic acid appeared after fermentation and increased after storage. This compound has also been described after the fermentation with *Lpb. plantarum* in citrus juice. The appearance of this compound seems to be related to the presence of phenylalanine and the action of *Lpb. plantarum* [43]. Besides, it has demonstrated antimicrobial activity against both Gram-positive and Gram-negative bacteria, such as *L. monocytogenes* and *Staphylococcus aureus* [44]. Hydroxyphenyllactic acid has also been described as a specific metabolite produced by *Lpb. plantarum* [45]. In fact, it increased during fermentation and storage. This compound has also shown antimicrobial activity against different pathogens, including *E. coli*.

Compound 11, dihydro-p-coumaric acid, has been described to increase after the fermentation of different nuts, such as almonds [46]. In fact, its concentration augmented more than four times after fermentation, and it remained stable after storage. Dihydrophaseic acid-ribofuranosyl-glucopyranoside has previously been described in cacao germs as a metabolite of abscisic acid produced during seed maturation and germination [47]. Besides, it has been hypothesized that fermentation contributes to the increase of glycosyl-conjugated dihydrophaseic acid forms because dihydrophaseic acid could be initially bound to cell-wall polysaccharides via esters or a glycosidic bond (un-extractable) and then released via fermentation [48].

p-Coumaric acid hexoside pentoside isomers I and II were identified at *m*/*z* 457, molecular formula C_20_H_26_O_12_ and fragments 119/163, as previously described by other authors [49]. Isomer I decreased after fermentation, whereas isomer II increased; this fact can be due to the influence of fermentation, which can affect the breaking of sugar bonds as well as the release of compounds bound to cell walls.

The other compounds were not affected or showed a decrease after fermentation, in accordance with previous studies which reported a decrease of glycosylated phenolic compounds after fermentation due to hydrolysis reactions [50].

**Table 4:** Identification of phenolic and other polar compounds.

Nº	Compounds	RT	Molecular Formula	*m/z* Experimental	*m/z* Calculated	Error (ppm)	Fragments	Reference
1	Phenyllactic acid	2.620	C_8_H_8_O_2_	135.045	135.045	−0.7	-	[51]
2	Hydroxyphenyllactic acid	2.640	C_9_H_10_O_4_	181.051	181.050	3.9	163 (C_9_H_7_O_3_)	[52]
3	Vanillic acid	2.725	C_8_H_8_O_4_	167.034	167.034	−0.6	-	[53]
4	Protocatechualdehyde-glucoside-xyloside	3.091	C_18_H_24_O_12_	431.119	431.119	0.2	137(C_7_H_6_O_3_ protocatechualdehyde)	[54]
5	Protocatechuic acid	3.459	C_7_H_6_O_4_	153.019	153.019	3.9	-	[54]
	Amygdalin hexoside	4.336	C_26_H_37_NO_16_	618.199	618.203	−4.8		[55]
6	Dehydrophaseic acid hexoside	4.746	C_21_H_32_O_10_	443.191	443.192	−1.6	-	[52]
7	Feruloyl-dihexoside isomer I	4.982	C_22_H_3_0O_14_	517.155	517.156	−1	193	[56]
8	Feruloyl-dihexoside isomer II	5.069	C_22_H_30_O_14_	517.156	517.156	0.8	193	[56]
9	Catechin	5.069	C_15_H_14_O_6_	289.071	289.071	−0.3	-	[53]
10	Prunasin pentoside	5.069	C_19_H_26_NO_10_	426.139	426.140	−2.1		[55]
11	Dihydro-p-coumaric acid	5.159	C_9_H_10_O_3_	165.056	165.055	4.8	353(C_20_H_18_O_6_)	[46]
12	Dihydrophaseic acid-ribofuranosyl-glucopyranoside	5.209	C_26_H_40_O_14_	575.232	575.234	−3	-	[47]
13	*p*-coumaric acid hexoside pentoside isomer I	5.437	C_20_H_26_O_12_	457.134	457.135	−0.9	163/119	[49]
14	*p*-coumaric acid hexoside pentoside isomer II	5.577	C_20_H_26_O_12_	457.134	457.135	−1.3	163/119	[49]
15	3-hydroxyphloretin-pentosylhexoside	5.668	C_26_H_32_O_15_	583.166	583.166	−0.3	289 (C_15_H_14_O_6_)	[57]
16	Ferulic acid derivative isomer I	5.761	C_21_H_28_O_13_	487.146	487.145	1.2	193 (C_10_H_10_O_4_)	[56]
17	Ferulic acid derivative isomer II	6.092	C_21_H_28_O_13_	487.145	487.145	0.4	193	[56]
18	*p*-coumaric acid hexoside pentoside derivative	6.208	C_24_H_38_O_12_	517.228	517.229	−1.2	163 (coumaric acid)/457 (p-coumaric acid hexoside pentoside)	[56]
19	Epicatechin	6.516	C_15_H_14_O_6_	289.071	289.071	0.3	-	[53]
20	Dihydroferulic acid derivative	6.723	C_21_H_32_O_12_	475.182	475.182	0.2	371/343(C_16_H_24_O_8_)/195 (C_10_H_12_O_4_)/181/166	-
21	Ferulic acid tripentoside derivative	6.910	C_25_H_34_O_16_	589.170	589.177	−12.4	-	[58]
22	Vanillin hexoside pentoside	6.963	C_19_H_26_O_13_	461.130	461.129	2	167/149	-
23	Dihydro sinapic acid hexoside-pentoside	7.534	C_24_H_40_O_12_	519.244	519.244	−0.6	387	[59]
24	(epi)-Catechin gallate	10.363	C_22_H_18_O_10_	441.085	441.082	2.9	289	[60]
25	Rutin	11.009	C_27_H_30_O_16_	609.146	609.146	0.3	447/301/300	[53]
26	Quercetin hexoside	11.265	C_21_H_20_O_12_	463.130	463.124	13.8	300/301/271/255	[53]
27	Kaempferol derivative	11.935	C_21_H_36_O_11_	463.218	463.218	1.1	285 (C_15_H_10_O_6_)	[53]
28	Isorhamnetin-3-O-rutinoside	12.026	C_28_H_32_O_16_	623.163	623.161	3	315 (C_16_H_12_O_7_)	[53]
29	Kaempferol-hexoside	12.308	C_21_H_20_O_11_	447.091	447.093	−3.4	285	[53]
30	Isorhamnetin-glucoside	12.362	C_22_H_22_O_12_	477.107	477.103	8.2	-	[61]
31	Quercetin-3-O-rhamnoside	12.525	C_21_H_20_O_11_	447.092	447.093	−0.9	300/301 (C_15_H_9_O_7_)/271/255	[52]

**Table 5:** Phenolic and other polar compounds quantified in samples produced in the control pilot scale during fermentation and after 15 days of storage at 4 °C determined by UPLC-MS. Data are expressed as µg/g of dry weight (d.w.).

Compounds	Sampling Times		
	Fermentation		Storage (4 °C)
	0 h	18 h	15 Days
Phenyllactic acid	nd	0.62 (±0.01)	0.901 (±0.001)
Hydroxyphenyllactic acid	0.86 (±0.05)	29.99 (±2.01)	39.36 (±0.37)
Vanillic acid	1.08 (±0.06)	<LOQ ^1^	<LOQ
Protocatechualdehyde-glucoside-xyloside	5.27 (±0.52)	2.42 (±0.32)	2.92 (±0.39)
Protocatechuic acid	0.27 (±0.13)	<LOQ	<LOQ
Dehydrophaseic acid hexoside	16.28 (±1.24)	9.64 (±0.23)	7.17 (±0.22)
Feruloyl-dihexoside isomer I	1.25 (±0.02)	<LOQ	nd
Feruloyl-dihexoside isomer II	2.00 (±0.33)	<LOQ	nd
Catechin	0.62 (±0.01)	0.428 (±0.007)	0.14 (±0.01)
Dihydro-p-coumaric acid	0.51 (±0.01)	2.27 (±0.02)	2.44 (±0.27)
Dihydrophaseic acid-ribofuranosyl-glucopyranoside	1.17 (±0.03)	2.65 (±0.1)	2.41 (±0.24)
p-coumaric acid hexoside pentoside isomer I	2.09 (±0.03)	1.27 (±0.04)	0.87 (±0.03)
p-coumaric acid hexoside pentoside isomer II	0.50 (±0.01)	0.836 (±0.005)	0.56 (±0.01)
3-hydroxyphloretin-pentosylhexoside	0.36 (±0.01)	0.229 (±0.005)	0.225 (±0.002)
Ferulic acid derivative isomer I	6.55 (±0.04)	2.62 (±1.24)	2.59 (±0.03)
Ferulic acid derivative isomer II	8.91 (±0.02)	8.45 (±0.07)	4.66 (±0.41)
p-coumaric acid hexoside pentoside derivative	0.29 (±0.01)	0.2332 (±0.0003)	0.256 (±0.005)
Epicatechin	0.18 (±0.03)	<LOQ	<LOQ
Dihydroferulic acid derivative	4.22 (±0.30)	2.76 (±0.13)	2.01 (±0.06)
Ferulic acid tripentoside derivative	0.26 (±0.11)	0.08 (±0.01)	<LOQ
Vanillin hexoside pentoside	0.94 (±0.01)	<LOQ	<LOQ
Dihydro sinapic acid hexoside-pentoside	0.461 (±0.002)	0.336 (±0.001)	nd
(epi)-Catechin gallate	<LOQ	<LOQ	<LOQ
Rutin	<LOQ	<LOQ	nd
Quercetin hexoside	<LOQ	<LOQ	nd
Kaempferol derivative	<LOQ	<LOQ	<LOQ
Isorhamnetin-3-O-rutinoside	<LOQ	<LOQ	nd
Kaempferol-hexoside	<LOQ	<LOQ	<LOQ
Isorhamnetin-glucoside	<LOQ	<LOQ	nd
Quercetin-3-O-rhamnoside	<LOQ	<LOQ	<LOQ
Total	54.07 (±1.56)	64.21 (±1.29)	65.61 (±1.21)

^1^: LOQ (µg/mL) were the following: vanillic acid 1.57, ferulic acid 0.89, quercetin 0.21, rutin 0.14, catechin 0.09. nd: not determined.

## 4. Conclusions

The findings of the present study can contribute to studying the risk assessment of plant-based fermented products obtained as cheese alternatives from a non-conventional raw material, such as nuts. Although other fermented vegetable products (i.e., olives, sauerkraut, etc.) have been deeply characterized, there are few data about the fermentative processes of this kind of matrices. This fact is even more important considering that these vegan foods are usually consumed without cooking, making contamination by pathogens a potential public health issue. The results of the laboratory-scale challenge tests demonstrated that, even though the growth of *L. monocytogenes*, *E. coli* and *S.* Enteritidis was not supported during process and storage, they can survive for a long period in this stressful environment with low pH. The data of the optimization production demonstrated that the application of a heat treatment of nuts in water at 85 °C for 10 min contributes to reduce the pathogen cell load, together with the use of the best performing starter strains and strict good manufacturing practices.

*Leuconostoc carnosum* 4010 and *Lpb. plantarum* 82, being bioprotective LAB strains, demonstrated to be the best starter combination for assuring the safety of the spreadable nut-fermented product. In addition, VOCs profiles showed the presence of important aroma compounds, and the total content of phenolic compounds increased after fermentation, without significant variations after 15 days in storage. Most compounds responsible for the increase have demonstrated antimicrobial activity against different pathogens and, thus, could be related to the improvement of the shelf life of the final product.

The use of this optimized starter microbial consortium, endowed with bioprotective features, allowed the set-up of a guided fermentation process, able to confer to the final cheese analogue aromatic, functional, and chemico-physical features. The development of a new vegan fermented product prototype suitable also for further industrial advancement can promote an increased availability of meat alternatives for consumers, avoiding the risk of outbreaks due to foodborne pathogens.

## Figures and Tables

**Table 1:** Inhibition halo (expressed as diameter in mm) of LAB strains or their supernatants against *L. monocytogenes* Scott A, *E. coli* 555 and *S.* Enteritidis 155.

Target Strain	Spot Cultures			
	***Leuc. mesenteroides*** ***LmV1***	***P. pentosaceus*** **PpV1**	***Lpb. plantarum*** **82**	***Leuc. carnosum*** **4010**
*L. monocytogenes* Scott A	14	14	13	15
*E. coli* 555	11	12	14	11
*S.* Enteritidis 155	12	12	15	12
	Neutralized cell-free supernatants			
*L. monocytogenes* Scott A	- ^1^	-	-	13
*E. coli* 555	-	-	-	-
*S.* Enteritidis 155	-	-	-	-
	Neutralized cell-free supernatants heat treated at 98 °C for 20 min			
*L. monocytogenes* Scott A	-	-	-	12
*E. coli* 555	-	-	-	-
*S.* Enteritidis 155	-	-	-	-
	Neutralized cell-free supernatants treated with pepsin			
*L. monocytogenes* Scott A	-	-	-	10
*E. coli* 555	-	-	-	-
*S.* Enteritidis 155	-	-	-	-
	Neutralized cell-free supernatants treated with proteinase K			
*L. monocytogenes* Scott A	-	-	-	-
*E. coli* 555	-	-	-	-
*S.* Enteritidis 155	-	-	-	-

^1^: No inhibition activity.

**Table 2:** Microbial counts (log CFU/g) and pH values of samples obtained in challenge test in laboratory-scale productions. Analyses were performed on nuts before and after soaking, on pre-fermented cream and during fermentation (25 °C) and product storage (4 °C). Standard deviations are reported in brackets. Starter strains: trial 1, *Leuc. mesenteroides* LmV1 and *P. pentosaceus* PpV1; trial 2, *Leuc. mesenteroides* LmV1 and *Lpb. plantarum* 82; trial 3, *Leuc. carnosum* 4010 and *Lpb. plantarum* 82.

	Sampling Time		pH ^1^	a_w_ ^1^	LAB ^1^	*Enterobacteriaceae* ^1^	*L. monocytogenes* Scott A	*E. coli* 555	*S.* Enteritidis 155
Trial 1	Soaking	0 h	6.51 (±0.04)	n.d. ^3^	7.50 (±0.08)	0.31 (±0.31)	5.61 (±0.06)	5.05 (±0.08)	5.55 (±0.06)
		10 h	4.91 (±0.02)	n.d.	8.97 (±0.05)	- ^2^	5.57 (±0.10)	5.11 (±0.05)	5.38 (±0.11)
	Product during fermentation/storage	0 h	6.04 (±0.03)	0.985 (±0.003)	7.87 (±0.11)	2.48 (±0.19)	4.51 (±0.12)	4.43 (±0.13)	4.20 (±0.12)
		24 h	4.67 (±0.02)	0.982 (±0.003)	9.26 (±0.09)	1.18 (±0.25)	4.68 (±0.05)	4.35 (±0.18)	4.05 (±0.09)
		48 h	4.41 (±0.01)	0.981 (±0.004)	9.20 (±0.08)	2.90 (±0.11)	4.71 (±0.11)	4.41 (±0.06)	4.10 (±0.15)
		7 d	4.44 (±0.02)	0.978 (±0.002)	9.55 (±0.07)	2.15 (±0.04)	4.62 (±0.14)	4.16 (±0.07)	3.70 (±0.13)
		15 d	4.61 (±0.02)	0.977 (±0.003)	9.42 (±0.10)	-	3.98 (±0.05)	3.36 (±0.09)	3.01 (±0.08)
		21 d	4.58 (±0.03)	0.979 (±0.002)	9.22 (±0.03)	-	3.66 (±0.08)	2.91 (±0.14)	2.52 (±0.11)
Trial 2	Soaking	0 h	6.53 (±0.03)	n.d.	7.45 (±0.16)	0.71 (±0.33)	5.82 (±0.08)	5.21 (±0.10)	5.34 (±0.18)
		10 h	4.85 (±0.01)	n.d.	9.15 (±0.13)	-	5.79 (±0.11)	5.16 (±0.06)	5.21 (±0.16)
	Product during fermentation/storage	0 h	5.93 (±0.02)	0.988 (±0.003)	8.05 (±0.05)	2.97 (±0.18)	4.29 (±0.13)	4.43 (±0.07)	4.22 (±0.08)
		24 h	4.41 (±0.03)	0.987 (±0.001)	9.11 (±0.09)	2.29 (±0.11)	1.90 (±0.06)	4.06 (±0.04)	3.80 (±013)
		48 h	4.35 (±0.04)	0.984 (±0.003)	9.21 (±0.08)	-	1.78 (±0.07)	4.02 (±0.07)	3.63 (±0.06)
		7 d	4.25 (±0.02)	0.983 (±0.002)	8.84 (±0.11)	-	1.62 (±0.18)	3.92 (±0.05)	3.54 (±0.12)
		15 d	4.40 (±0.01)	0.980 (±0.003)	8.97 (±0.12)	-	Absent in 25 g	2.63 (±0.11)	2.12 (±0.05)
		21 d	4.34 (±0.03)	0.978 (±0.004)	8.78 (±0.09)	-	Absent in 25 g	-	Absent in 25 g
Trial 3	Soaking	0 h	6.39 (±0.02)	n.d.	7.63 (±0.09)	0.80 (±0.25)	5.90 (±0.10)	5.10 (±0.12)	5.29 (±0.18)
		10 h	4.61 (±0.02)	n.d.	9.05 (±0.07)	-	5.68 (±0.08)	5.25 (±0.15)	5.35 (±0.14)
	Product during fermentation/storage	0 h	5.53 (±0.02)	0.984 (±0.003)	7.75 (±0.15)	1.96 (±0.25)	5.61 (±0.15)	5.34 (±0.07)	5.22 (±0.13)
		18 h	4.33 (±0.04)	0.981 (±0.001)	9.20 (±0.12)	0.33 (±0.33)	2.74 (±0.06)	5.67 (±0.09)	5.46 (±0.09)
		7 d	4.08 (±0.02)	0.983 (±0.002)	9.01 (±0.16)	-	Absent in 25 g	5.35 (±0.22)	5.31 (±0.13)
		15 d	4.13 (±0.03)	0.980 (±0.003)	8.73 (±0.05)	-	Absent in 25 g	2.45 (±0.05)	2.16 (±0.07)
		21 d	4.22 (±0.03)	0.977 (±0.003)	8.80 (±0.13)	-	Absent in 25 g	0.33 (±0.33)	Absent in 25 g

^1^: data referred to the control not inoculated with pathogens; ^2^: under detection limit (0.33 log CFU/g); ^3^: not determined.

**Table 3:** Volatile compounds detected in the control pilot-scale production before and after the fermentation and after 15 and 30 days of storage at 4 °C, as determined by SPME-GC–MS. Data are expressed as ratio between peak area of each molecule and peak area of the internal standard (4-methyl-2-pentanol), and standard deviations are reported in brackets.

Aroma Compounds		Sampling Times			
	ID ^1^	Fermentation		Storage (4 °C)	
		0 h	18 h	15 Days	30 Days
2,3-Butanedione	MS, RF	- ^2^	0.54 (±0.04)	2.25 (±0.04)	1.55 (±0.19)
2-Heptanone	MS	-	1.55 (±0.11)	1.18 (±0.28)	1.25 (±0.24)
3-Hydroxy-2-butanone	MS, RF	-	0.75 (±0.09)	29.19 (±1.37)	31.31 (±1.12)
2-Nonanone	MS	-	-	1.37 (±0.16)	1.64 (±0.22)
Total ketones		-	2.84	33.99	35.75
3-Methyl-butanal	MS, RF	0.47 (±0.10)	1.47 (±0.25)	0.66 (±0.03)	0.92 (±0.22)
Hexanal	MS, RF	1.16 (±0.09)	2.28 (±0.10)	2.54 (±0.11)	1.77 (±0.30)
Nonanal	MS	3.14 (±0.28)	2.39 (±0.05)	2.51 (±0.18)	2.54 (±0.42)
Benzaldehyde	MS, RF	111.09 (±5.61)	101.35 (±3.55)	32.78 (±2.41)	22.87 (±4.87)
Total aldehydes		115.86	107.49	38.49	28.10
Ethyl alcohol	MS, RF	47.84 (±0.18)	92.49 (±4.55)	86.95 (±5.60)	101.52 (±3.41)
3-Methyl-2-butanol	MS	4.90 (±0.18)	3.39 (±0.15)	3.80 (±0.04)	6.17 (±0.35)
1-Pentanol	MS	2.21 (±0.30)	2.19 (±0.07)	1.00 (±0.10)	1.27 (±0.13)
1-Hexanol	MS, RF	12.53 (±1.27)	16.98 (±1.90)	13.58 (±1.01)	15.54 (±1.26)
1-Heptanol	MS	2.80 (±0.25)	3.23 (±0.36)	2.83 (±0.09)	3.59 (±0.18)
1-Octanol	MS, RF	2.53 (±0.20)	3.00 (±0.15)	3.37 (±0.12)	3.93 (±0.63)
Benzyl alcohol	MS, RF	9.69 (±0.42)	24.41 (±0.37)	35.21 (±1.04)	43.30 (±1.35)
Phenylethyl alcohol	MS, RF	6.15 (±0.40)	7.45 (±0.28)	6.93 (±0.37)	6.90 (±0.25)
Total alcohols		88.65	153.14	153.67	181.91
Acetic acid	MS, RF	6.96 (±0.22)	57.36 (±0.55)	88.64 (±1.01)	112.51 (±0.43)
Hexanoic acid	MS, RF	2.58 (±0.09)	5.98 (±1.33)	3.39 (±0.13)	3.72 (±0.30)
Total acids		9.54	63.34	92.03	116.23

^1^: ID, Reliability of identification; MS, tentative identification by mass spectrum; RF, mass spectrum and retention time identical with a reference compound. ^2^: Not detected.

## Data Availability

The original contributions presented in the study are included in the article, further inquiries can be directed to the corresponding author.
